# An IFN/STAT1/CYBB axis defines protective plasmacytoid DC–neutrophil crosstalk in *Aspergillus fumigatus*–infected mice

**DOI:** 10.1172/JCI190107

**Published:** 2025-08-05

**Authors:** Yahui Guo, Mariano A. Aufiero, Kathleen A.M. Mills, Simon A. Grassmann, Hyunu Kim, Mergim Gjonbalaj, Paul Zumbo, Audrey Billips, Katrina B. Mar, Yao Yu, Laura C. Echeverri Tirado, Lena Heung, Amariliz Rivera, Doron Betel, Joseph C. Sun, Tobias M. Hohl

**Affiliations:** 1 Infectious Disease Service, Department of Medicine,; 2 Human Oncology and Pathogenesis Program, and; 3 Louis V. Gerstner Jr. Graduate School of Biomedical Sciences, Sloan Kettering Institute, Memorial Sloan Kettering Cancer Center, New York, New York, USA.; 4 Immunology and Microbial Pathogenesis Graduate Program, Weill Cornell Graduate School, New York, New York, USA.; 5 Immunology Program, Sloan Kettering Institute, Memorial Sloan Kettering Cancer Center, New York, New York, USA.; 6 Applied Bioinformatics Core, Department of Physiology and Biophysics, Weill Cornell Medicine, New York, New York, USA.; 7 Center for Immunity and Inflammation, New Jersey Medical School, Rutgers–The State University of New Jersey, Newark, New Jersey, USA.; 8 Department of Medicine and Department of Biomedical Sciences, Women’s Guild Lung Institute, Cedars-Sinai Medical Center, Los Angeles, California, USA.; 9 Applied Bioinformatics Core, Division of Hematology and Medical Oncology, Department of Medicine, Institute for Computational Biomedicine, Weill Cornell Medicine, New York, New York, USA.

**Keywords:** Immunology, Infectious disease, Fungal infections, Neutrophils

## Abstract

*Aspergillus fumigatus* is the most common cause of invasive aspergillosis (IA), a devastating infection in immunocompromised patients. Plasmacytoid DCs (pDCs) regulate host defense against IA by enhancing neutrophil antifungal properties in the lung. Here, we define the pDC activation trajectory during *A*. *fumigatus* infection and the molecular events that underlie the protective pDC–neutrophil crosstalk. Fungus-induced pDC activation began after bone marrow egress and resulted in pDC-dependent regulation of lung type I and type III IFN levels. These pDC-derived products acted on type I and type III IFN receptor–expressing neutrophils and controlled neutrophil fungicidal activity and ROS production via STAT1 signaling in a cell-intrinsic manner. Mechanistically, neutrophil STAT1 signaling regulated transcription and expression of *Cybb*, which encodes one of 5 NADPH oxidase subunits. Thus, the results indicate that pDCs regulate neutrophil-dependent immunity against inhaled molds by controlling local expression of a subunit required for NADPH oxidase assembly and activity in the lung.

## Abstract 2

pDCs are essential for defense against Aspergillus fumigatus. Fungus-induced pDC activation in the lung results in type I and III IFN release which in turn regulates neutrophil STAT1-dependent Cybb transcription and translation. Thus, pDCs calibrate NADPH oxidase-dependent neutrophil effector activity at the portal of infection.

## Graphical abstract

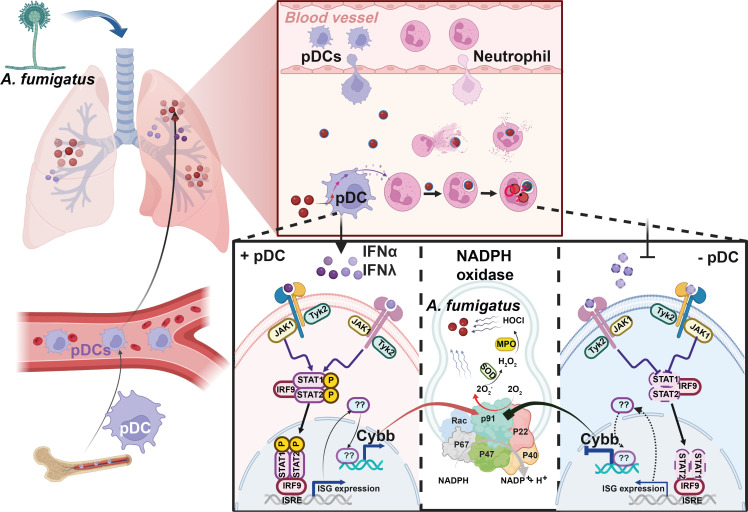

## Introduction

Invasive pulmonary aspergillosis, a life-threatening mold infection, occurs when the respiratory immune system fails to eradicate ubiquitous inhaled *Aspergillus* spores (i.e., conidia) prior to their germination into tissue-invasive hyphae (1). At-risk populations include patients with acute leukemia and other BM disorders; recipients of hematopoietic cell and lung transplants; individuals with immune-related or neoplastic diseases treated with prolonged corticosteroid therapy or with novel targeted biologics (e.g., ibrutinib) that blunt fungal immune surveillance pathways (2–4); and patients with severe respiratory virus infections, including influenza and COVID-19 (5, 6). As a result of these medical advances and global pandemic viruses, *A*. *fumigatus* has become the most common agent of mold pneumonia worldwide (6–12).

Host defense against airborne mold conidia depends on intact myeloid cell numbers and function at the respiratory mucosa. Lung-infiltrating neutrophils and monocyte-derived DCs (Mo-DCs) play essential roles in killing phagocytosed conidia, with a central role for products of NADPH oxidase in this process (3, 13–15). Patients with chronic granulomatous disease (CGD) and defective NADPH oxidase function are uniquely vulnerable to invasive aspergillosis (IA), with a lifetime prevalence of 40%–55% (16). Exposure to products of NADPH oxidase induces a regulated cell death process in conidia trapped within neutrophil phagosomes, resulting in sterilizing immunity at the respiratory mucosal barrier (17).

An emerging theme in host defense against *Aspergillus* is the essential role of intercellular crosstalk to license neutrophil effector properties in situ. On one hand, recruited monocytes and Mo-DCs promote neutrophil ROS production through type I and III IFN release, though the link between IFN production and neutrophil ROS activity remains undefined (15). On the other hand, we discovered that fungus-engaged neutrophils and Mo-DCs release CXCL9 and CXCL10, which results in recruitment of CXCR3^+^ pDCs by promoting their influx from the circulation into the lung. In the lung, pDCs enhance neutrophil fungicidal properties and are essential for host defense, even in the presence of lung-infiltrating monocytes and Mo-DCs (18). However, the underlying molecular mechanisms that regulate pDCs to neutrophil crosstalk remain undefined. Thus, both Mo-DCs and pDCs independently enhance neutrophil antifungal activity, but it is unknown whether pDCs employ similar or distinct mechanisms of intercellular crosstalk with neutrophils compared with monocytes and Mo-DCs. Critical open questions relate to the identity of pDC-derived molecules that are required for protective crosstalk with neutrophils and to the ensuing molecular changes in neutrophils that regulate antifungal activity in situ.

In this study, we demonstrate that pDCs represent a key and indispensable source of type I and type III IFNs in the lung during respiratory *A*. *fumigatus* infection. In turn, pDC-dependent and IFN-induced STAT1 signaling controlled neutrophil *Cybb* expression, which encoded an essential subunit of the NADPH oxidase complex. Thus, protective pDC–neutrophil crosstalk primarily harnessed intercellular IFN/STAT1 signaling to calibrate the synthesis of a critical component of the NADPH oxidase complex, thereby licensing neutrophils to achieve optimal antifungal activity and promoting sterilizing responses against inhaled mold spores.

## Results

### Aspergillus fumigatus induces pDC activation in the lung.

**Figure 1:**
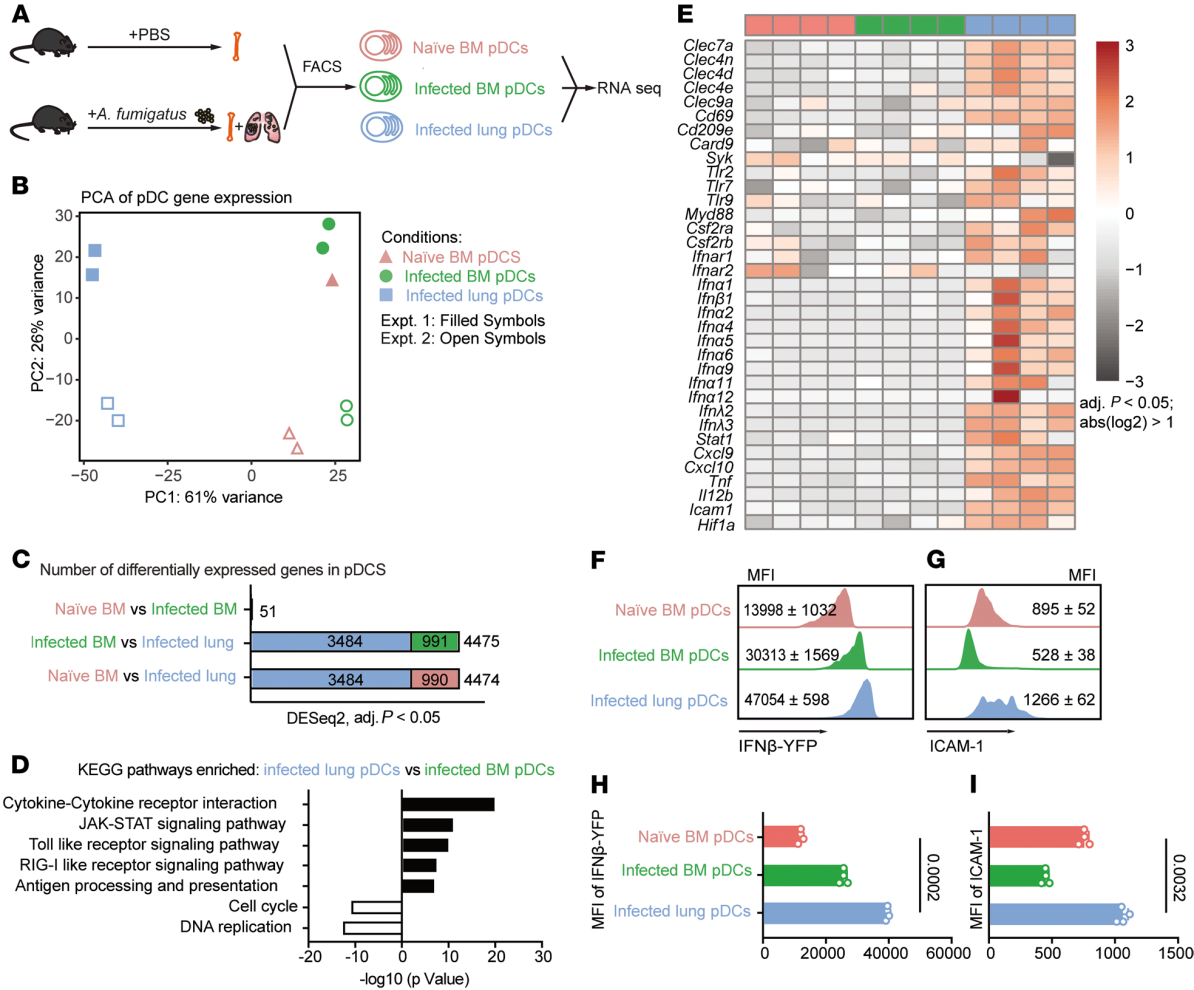
pDC transcriptome following *A*. *fumigatus* infection. (**A**) Experimental scheme for bulk RNA-Seq of BM pDCs sorted from naive mice (red symbols) and BM (green symbols) and lung (blue symbols) pDCs sorted from *A*. *fumigatus*–infected mice. i.n., intranasal. (**B**) Principal component analysis of gene expression in sorted BM pDCs from naive (triangles) and infected mice (circles) and of sorted lung pDCs from infected mice (squares). Each symbol represents a biological replicate. pDCs from 10 mice were pooled for each replicate, and 2 replicates were included in each of 2 experiments, denoted as Expt1 and Expt 2. (**C**) Number of DEGs for 3 comparisons of 2 pDC subsets, with blue indicating common DEGs and red and green indicating DEGs limited to a single comparison. (**D**) Differentially enriched KEGG pathways (*q* < 0.05) in pDCs isolated from infected lungs versus infected BM. Black bars indicate pathways enriched in lung pDCs from infected mice; white bars indicate pathways enriched in BM pDCs from infected mice. (**E**) Heatmap for 35 selected genes with >2 -fold difference in expression and FDR *P* < 0.05. Each lane represents 1 replicate from 2 experiments. (**F**–**I**) Representative flow cytometry plots (**F** and **G**) and quantified MFI (**H** and **I**) of IFN YFP and ICAM1 expression in pDCs isolated from naive BM, the BM of *A*. *fumigatus*–infected mice, or BM from the lungs of *A*. *fumigatus*–infected mice. (**A**–**I**) Infection dose: 3 × 10^7^ CEA10 conidia via intratracheal route; analysis 72 hpi. **B**–**E**: Data were pooled from 2 independent experiments. **H** and **I**: Statistical analysis: Kruskal-Wallis test. See also Supplemental Figure 1.

In response to respiratory fungal infection, pDCs exit the BM, enter the circulation, and traffic to the lung. To gain an understanding of the pDC activation trajectory during this infection-induced trafficking event, we conducted an unbiased transcriptome analysis of BM pDCs isolated from uninfected mice and of BM and lung pDCs isolated from mice at 72 hours after infection (hpi) with *A*. *fumigatus* strain CEA10, since pDCs (18) enter the lung at this time point (Figure 1, A and B, and Supplemental Figure 1A; supplemental material available online with this article; https://doi.org/10.1172/JCI190107DS1). For each pDC RNA-Seq sample, we pooled sorted pDCs from 10 mice from each tissue examined and analyzed 4 biological replicates. The bulk pDC transcriptome was remarkably similar in naive and infected BM pDCs, with only 51 differentially expressed genes (DEGs), supporting a model in which pDC activation occurs en route to the site of infection. In these 2 groups of BM pDCs, we did not observe notable differences in the transcription of genes that encode cytokines or IFNs (Figure 1C and Supplemental Figure 1B). In contrast, RNA-Seq analysis revealed significant changes in the pDC transcriptome when lung pDCs were isolated from *Aspergillus*-infected mice and compared with BM pDCs (Figure 1B). Overall, pDCs from infected lungs had 4,475 and 4,474 DEGs compared with pDCs isolated from the BM of naive and infected mice, respectively. Among these, there were 3,484 common DEGs, with an additional 990 and 991 DEGs, respectively, that were restricted to a single comparison (Figure 1C and Supplemental Figure 1, C and D). Using Kyoto Encyclopedia of Genes and Genomes (KEGG) pathway enrichment analysis, we found that pathways involved in the cytokine-cytokine receptor interaction and Toll-like receptor, RIG-I, and JAK/STAT signaling were among the most upregulated pathways in pDCs isolated from infected lungs compared with BM pDCs isolated from the same mice (Figure 1D and Supplemental Figure 1E).

Lung-infiltrating pDCs upregulated genes implicated in fungal recognition, including the C-type lectin receptors *Clec7a*, *Clec4n*, *Clec4d*, *Clec4e*, *Clec9a*, *Cd69*, and *Cd209e* and downstream signaling molecules (*Card9* and *Syk*). pDCs upregulated Toll-like (*Tlr2*, *Tlr7*, *Tlr9*, and *Myd88*) and growth factor (*Csf2ra*, *Csf2rb*) signaling pathways as well. Notably, we found that expression of type I IFN, type III IFN, and the type I IFN receptor (*Ifnar1* and *Ifnar2*) was markedly increased in lung-infiltrating pDCs isolated from infected mice (Figure 1E), consistent with prior reports that *A*. *fumigatus* infection induces type I and type III IFN release in the lung (15). In addition, lung-infiltrating pDCs upregulated cytokine and chemokine (*Cxcl9*, *Cxcl10*, and *Il12b*), integrin receptor (i.e., *Icam1* [intercellular adhesion molecule]) and *Hif1a* mRNAs (Figure 1E).

To examine the impact of pDC transcriptional changes on protein expression, we infected IFN-β reporter mice with *A*. *fumigatus* and found that *Ifnb* promoter–driven fluorescent protein expression increased when BM pDCs trafficked to the lung (Figure 1, F and H). Similarly, we observed pDC trafficking-dependent increases in ICAM1 surface expression (Figure 1, G and I). Thus, *A*. *fumigatus* infection substantially altered the pDC transcriptome at the portal of infection.

### pDCs regulate type I and type III IFN in the lung during A. fumigatus infection.

**Figure 2:**
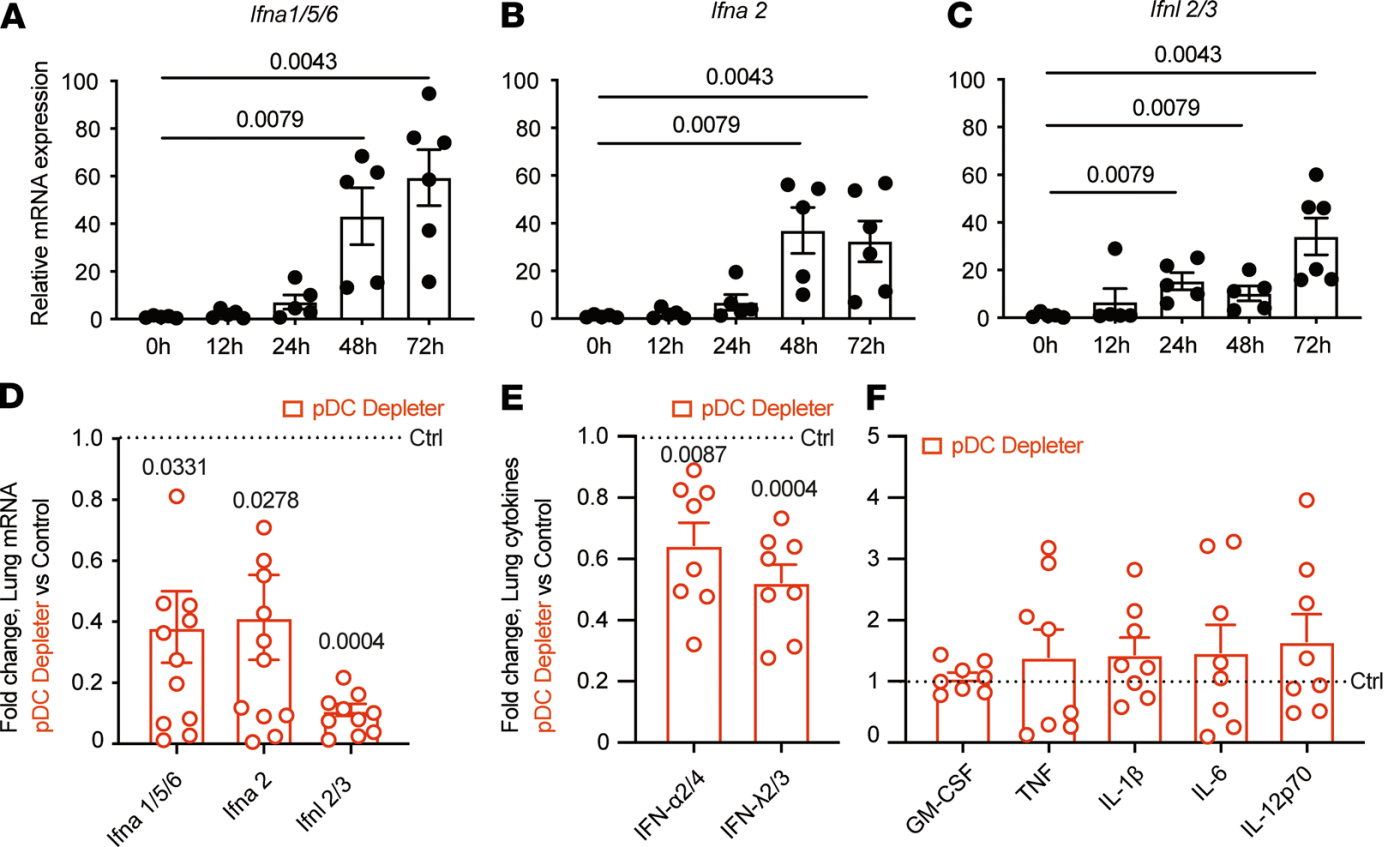
Cytokine profiles in pDC-depleted mice during *A*. *fumigatus* infection. (**A**–**C**) *Ifn* gene expression, measured by qRT-PCR using TaqMan probes, in the lung of C57BL6/J mice at indicated times; *n* = 5–6 per group. (**D**) Lung *Ifn* gene expression and (**E** and **F**) lung cytokine levels measured by ELISA in DT-treated pDC depleter mice (BDCA2-DTR^Tg/+^) and DT-treated non-Tg littermate controls (dotted line); *n* = 8–11 per group. **A**–**F**: Infection dose: 3 × 10^7^ CEA10 conidia via intratracheal route; analysis 72 hpi. Data were pooled from 2 independent experiments and presented as mean ± SEM. Data points represent individual mice. Statistical analysis: Kruskal-Wallis test.

To examine the contribution of pDCs to the lung inflammatory milieu, we examined type I (*Ifna1/2/5/6*) and type III (*Ifnl2/3*) IFN mRNA expression and found marked IFN induction between 48 and 72 hpi (Figure 1E and Figure 2, A–C), temporally coincident with lung pDC influx observed in a prior study (18). *Aspergillus*-induced lung type I and III IFN and pDC influx was observed following infection by different strains (i.e., CEA10 and Af293; Supplemental Figure 2, A and B), consistent with the idea that these features of the immune response are general and not strain specific.

To determine whether pDCs directly control type I and type III IFN induction, we infected BDCA2-DTR (pDC depleter) mice, in which diphtheria toxin (DT) administration specifically ablates pDCs at rest and under inflammatory conditions (19) but does not ablate CCR2-expressing monocytes and Mo-DCs, which have been implicated in type I and type III IFN release following *A*. *fumigatus* infection (15, 18). pDC depleter mice exhibited a 60%–80% reduction in lung *Ifna1/2/5/6* and *Ifnl2/3* mRNA levels at 72 hpi (Figure 2D), demonstrating that pDCs directly controlled the induction of type I and III IFN mRNA, consistent with the bulk RNA-Seq data and with their activation trajectory in the *Aspergillus*-infected lung (Figure 1E).

To determine the importance of pDCs for lung cytokine levels, we next compared lung cytokine profiles by ELISA in pDC-depleted mice and nontransgenic, co-housed littermate controls. pDC ablation resulted in a partial depletion (40%–60%) of lung type I (IFN-α2/4) and type III (IFN-λ2/3) IFN levels. In contrast, other proinflammatory cytokines implicated in pulmonary antifungal defense (e.g., GM-CSF ref. 20; TNF, ref. 21; IL-1β, ref. 22; IL-6, ref. 23; and IL-12, ref. 24) were not impacted by pDC ablation, due to their production by other cellular sources (12) in the lung (Figure 2, E and F). These data establish that pDCs played a critical role in regulating lung type I and type III IFN levels during *A*. *fumigatus* infection.

### STAT1 signaling in neutrophils controls intracellular killing of Aspergillus conidia.

Type I and type III IFNs both activate STAT1 signaling in target cells and are essential for host defense against *A*. *fumigatus* (15). Under baseline conditions and during *Aspergillus* infection, lung neutrophils expressed both type I IFN receptor and type III IFN receptor mRNA, as determined by RNAscope analysis of lung sections (Supplemental Figure 3, A and B) and RNA-Seq analysis of sorted lung neutrophils (Supplemental Figure 3C). Targeted ablation of *Stat1* in neutrophils renders mice susceptible to IA (15), yet it is unclear whether STAT1 signaling is required in a myeloid cell–intrinsic or –extrinsic manner and, in the case of the former possibility, whether STAT1 signaling regulates the transcription and translation of critical antifungal effector molecules.

**Figure 3:**
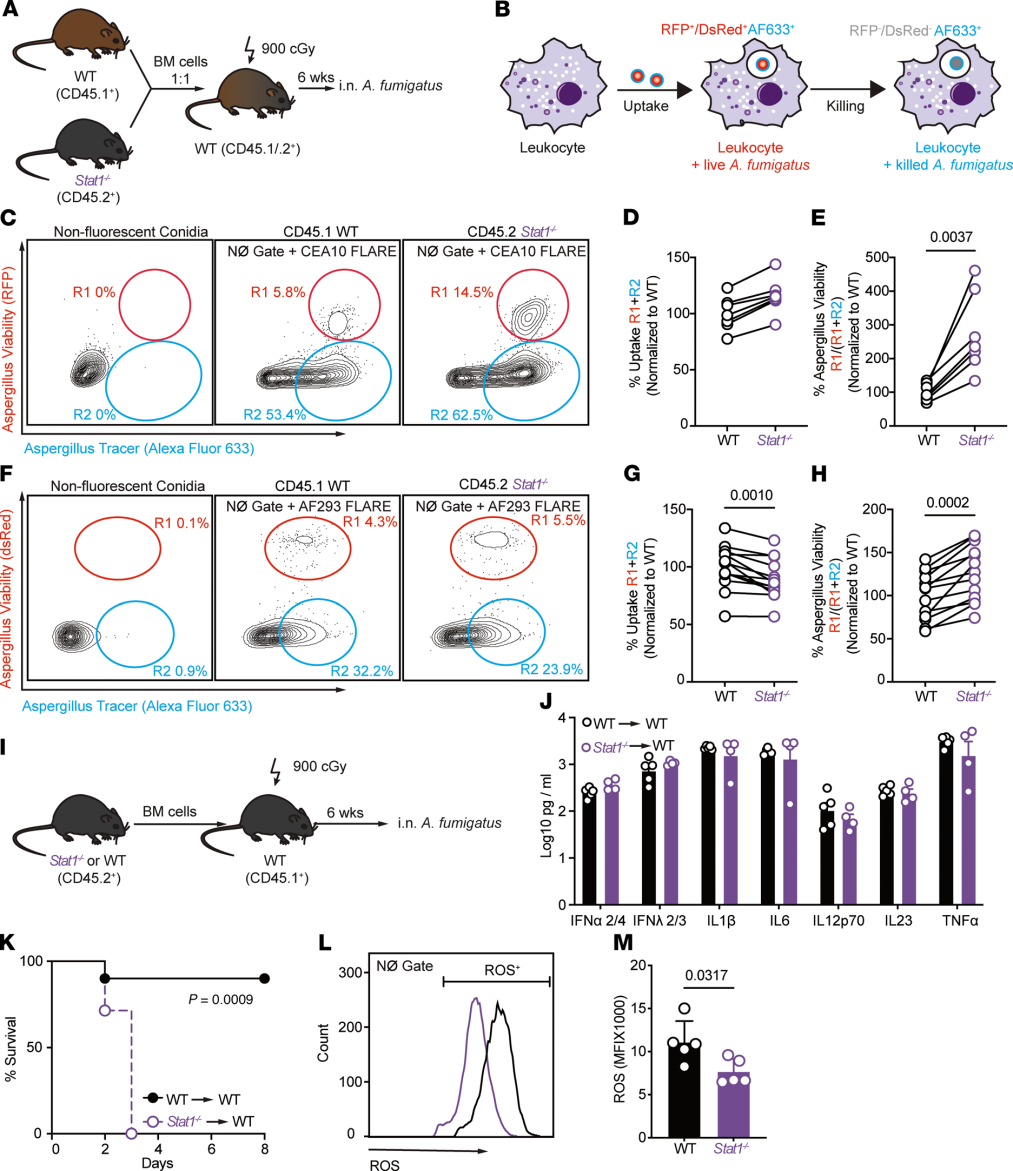
STAT1 signaling regulates neutrophil-intrinsic *Aspergillus* killing. (**A**) Scheme to generate *Stat1^–/–^* and *Stat1^+/+^* mixed bone marrow (BM) chimeric mice and (**B**) illustration of the 2-component FLARE system used to measure conidial uptake and killing by *Stat1^–/–^* and *Stat1^+/+^* lung leukocytes in mixed BM chimeric mice. (**C**) Representative plots showing CEA10-RFP and AF633 fluorescence intensity of lung neutrophils. The R1 gate denotes neutrophils that contain live conidia, the R2 gate denotes neutrophils that contain killed conidia. (**D** and **E**) The plots show (**D**) normalized lung neutrophil conidial uptake ([R1 + R2] ± SEM) and (**E**) conidial viability (R1/[R1 + R2] ± SEM) in indicated lung neutrophils isolated from *Stat1^–/–^* (purple symbols) and *Stat1^+/+^* (black symbols) mixed BM chimeric mice (*n* = 8 per group). (**F**) Representative plot showing DsRed and AF633 fluorescence intensity of lung neutrophils. The R1 gate denotes neutrophils that contain live conidia, the R2 gate denotes neutrophils that contain killed conidia. (**G** and **H**) The plots show (**G**) normalized lung neutrophil conidial uptake (R1 + R2) ± SEM and (**H**) conidial viability (R1/[R1 + R2] ± SEM) in indicated lung neutrophils isolated from *Stat1^–/–^* and *Stat1^+/+^* mixed BM chimeric mice (*n* = 7 per group). (**I**) Scheme to generate *Stat1^–/–^* and WT (*Stat1^+/+^*) chimeric mice. (**J**) Lung cytokine levels in *Stat1^–/–^*→WT (purple symbols) and *Stat1^+/+^*→WT single chimeric mice. (**K**) Kaplan-Meier survival curve of *Stat1^–/–^* and *Stat1^+/+^* chimeric mice (*n* = 7–8 per group) infected with 3 × 10^7^ to 6 × 10^7^ CEA10 conidia. (**L**) Representative plot and (**M**) mean ± SEM neutrophil ROS production in cells isolated from *Stat1^–/–^* →WT and *Stat1^+/+^*→WT single chimeric mice (*n* = 5 per group). **C**–**E**: Infection dose: 3 × 10^7^ CEA10 FLARE conidia via intratracheal route; analysis 72 hpi. **F**–**H**: Infection dose: 3 × 10^7^ Af293 FLARE conidia via intratracheal route; analysis 72 hpi. **J**–**M**: Infection dose: 3 × 10^7^ to 6 × 10^7^ CEA10 conidia via intratracheal route. **D**, **E**, **G**, and **H**: Data are representative of 2 experiments. Data points represent individual mice. Statistical analysis: paired *t* test. **K** and **M**: Data are representative of 2 experiments. Data points represent individual mice. Statistical analysis: Mann-Whitney *U* test. See also Supplemental Figures 3 and 4. NØ, neutrophil.

To distinguish these possibilities, we first generated mixed BM chimeric mice (1:1 mixture of CD45.2^+^ *Stat1*^−/−^ and CD45.1^+^ *Stat1^+/+^* donor BM cells → lethally irradiated CD45.1^+^ CD45.2^+^ *Stat1*^+/+^ recipients) and compared the fungicidal activity of *Stat1^–/–^* and *Stat1^+/+^* leukocytes within the same lung inflammatory context (Figure 3A). To accomplish this, we utilized fluorescent *Aspergillus* reporter (FLARE) conidia, which encode a red fluorescent protein (RFP; viability fluorophore) and are labeled with an Alexa Fluor 633 (AF633; tracer fluorophore) (25). FLARE conidia enabled us to distinguish live (RFP^+^AF633^+^) and dead (RFP^–^AF633^+^) conidia during leukocyte interactions with single encounter resolution (Figure 3B). For these experiments, we utilized both Af293- and CEA10-derived FLARE strains.

Following infection with CEA10-FLARE conidia, we measured the frequency of neutrophil fungal uptake (Figure 3C; frequency of neutrophil uptake = R1 + R2) and proportion of fungus-engaged neutrophils that contained live conidia (Figure 3C; proportion of fungus-engaged neutrophils with live conidia = R1/[R1 + R2]). *Stat1* deficiency did not affect lung neutrophil fungal uptake at 72 hpi compared with *Stat1^+/+^* neutrophils in the same lung (Figure 3D). However, the frequency of fungus-engaged neutrophils that contained live conidia was higher in *Stat1*^−/−^ neutrophils than in *Stat1^+/+^* neutrophils (Figure 3E). We confirmed these findings with the Af293 FLARE strain (Figure 3, F–H). Thus, neutrophil-engulfed conidia were more likely to be viable in *Stat1^–/–^* neutrophils than in *Stat1^+/+^* neutrophils in the same lung tissue environment (Figure 3, E and H), indicating a cell-intrinsic function for STAT1 in neutrophil antifungal activity.

To examine whether STAT1 signaling in hematopoietic cells impacts lung cytokine levels, we compared lung cytokine profiles in mice that lacked *Stat1* in radiosensitive hematopoietic cells and found no difference in lung IFN-α2/4, IFN-λ2/3, IL-1β, IL-6, IL-12p70, IL-23, and TNF levels (Supplemental Figure 3, I and J, and Supplemental Figure 4A). As expected, mice that lacked *Stat1* in radiosensitive hematopoietic cells were more susceptible to *A*. *fumigatus* challenge than mice with *Stat1* sufficiency in the same compartment (Figure 3K).

We found in our previous work that lung pDCs regulate neutrophil ROS generation during respiratory *A*. *fumigatus* challenge (18). We measured neutrophil ROS production in *Stat1^+/+^* and *Stat1*^−/−^ neutrophils and found that the ROS mean fluorescence intensity (MFI) in *Stat1*^−/−^ ROS^+^ lung neutrophils was significantly reduced compared with *Stat1^+/+^* ROS^+^ lung neutrophils at 72 hpi (Figure 3, L and M). We enumerated myeloid cell infiltration in *Stat1^+/+^* and *Stat1*^−/−^ lungs at 72 hpi and found no difference in the recruitment of neutrophils, monocytes, and pDCs in *Stat1^–/–^* mice. There was a slight decrease in Mo-DC numbers in *Stat1^–/–^* mice, which suggests that monocytes may exhibit a limited differentiation into Mo-DCs (26), resulting in reduced lung Mo-DC numbers in *Stat1*^−/−^ mice compared with control mice (Supplemental Figure 4, B–E). Collectively, these experiments indicate that STAT1 acts in a neutrophil-intrinsic manner to regulate antifungal effector activity and that STAT1 deficiency in radiosensitive cells or global STAT1 deficiency do not cause marked alterations in key lung cytokine mediators (IFN-α2/4, IFN-λ2/3, IL-1β, IL-6, IL-12p70, IL-23, and TNF) (22, 24, 27–30) implicated in host defense or in myeloid cell recruitment and survival in the infected lung.

### pDCs regulate neutrophil STAT1–dependent antifungal activity.

**Figure 4:**
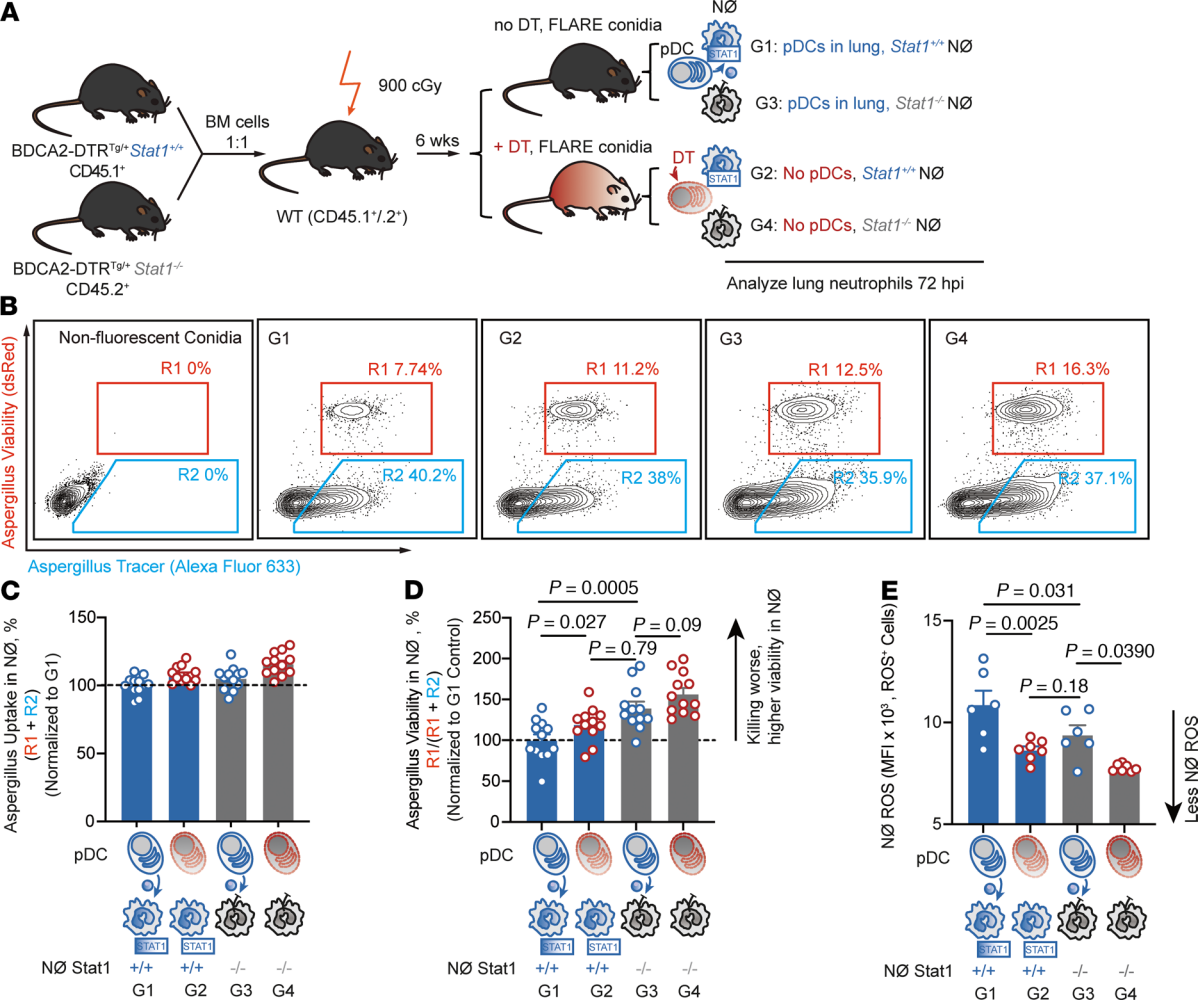
pDCs regulate neutrophil STAT1–dependent antifungal activity. (**A**) Scheme to generate and to deplete pDCs or not in CD45.1^+^ BDCA2- DTR^Tg/+^ *Stat1^+/+^* and CD45.2^+^BDCA2^–^DTR^Tg/+^ *Stat1^–/–^* mixed BM chimeric mice, resulting in 4 experimental groups (G1–G4). (**B**–**D**) Representative plots (**B**) display DsRed and AF633 fluorescence intensity in lung neutrophils from 4 experimental groups: G1, pDC^+^ *Stat1^+/+^* (*Stat1^+/+^* neutrophils from pDC-sufficient mice); G2, pDC^–^ *Stat1^+/+^* (*Stat1^+/+^* neutrophils from pDC-depleted mice); G3, pDC^+^ *Stat1^–/–^* (*Stat1^–/–^* neutrophils from pDC-sufficient mice); G4, pDC^–^ *Stat1^–/–^* (*Stat1^–/–^* neutrophils from pDC-depleted mice). The R1 gate indicates the frequency of neutrophils that contain live conidia; gate R2 indicates the frequency of neutrophils that contain killed conidia. (**C** and **D**) The plots show mean neutrophil (**C**) conidial uptake (R1 + R2) ± SEM and (**D**) conidial viability (R1/[R1 + R2]) ± SEM in indicated lung neutrophils isolated from the 4 groups. *n* = 12 per group. (**E**) Mean ± SEM ROS production in indicated lung neutrophils; *n* = 6 per group. **B**–**D**: Infection dose: 3 × 10^7^ Af293 FLARE conidia via intratracheal route; analysis 72 hpi. (**C** and **D**) Data were pooled from 2 independent experiments and presented as mean ± SEM, (**E**) Data are representative of 2 experiments. Data points represent individual mice. Statistical analysis: Kruskal-Wallis test. See also Supplemental Figure 3.

To explore whether pDCs regulate neutrophil STAT1–dependent antifungal activity, we bred CD45.2^+^ BDCA2-DTR^Tg/+^ mice with CD45.2^+^ *Stat1^–/–^* mice to generate CD45.2^+^ BDCA2-DTR^Tg/+^ *Stat1^–/–^* mice and bred BDCA2-DTR^Tg/+^ *Stat1^+/+^* mice to the CD45.1^+^ (C57BL6.SJL) background. BM cells from both strains were mixed in a 1:1 ratio and utilized as donor cells to generate mixed BM chimeric mice (CD45.1^+^ BDCA2-DTR^Tg/+^ *Stat1^+/+^* and CD45.2^+^ BDCA2-DTR^Tg/+^ *Stat1^–/–^*→CD45.1^+^CD45.2^+^ recipient mice). This experimental design enabled us to compare the fungicidal activity of *Stat1^+/+^* and *Stat1^–/–^* neutrophils in the same lung, in either the absence or presence of pDCs, through administration or omission of DT to mixed BM chimeric mice (Figure 4A).

This experimental approach yielded 4 groups of neutrophils that were analyzed 72 hpi with FLARE conidia. Group 1 (G1) neutrophils were *Stat1^+/+^* neutrophils isolated from pDC-sufficient mice; G2 neutrophils were *Stat1^+/+^* neutrophils isolated from pDC-ablated mice; G3 neutrophils were *Stat1^–/–^* neutrophils isolated from pDC-sufficient mice; and G4 neutrophils were *Stat1^–/–^* neutrophils isolated from pDC-ablated mice (Figure 4A). There was no difference in conidial uptake among these 4 groups of neutrophils (Figure 4, B and C), indicating that pDCs do not control neutrophil conidial uptake and that neutrophil-intrinsic STAT1 signaling is dispensable for this process.

The frequency of neutrophils that contained live conidia was markedly increased in G3 (pDC^+^ lungs; *Stat1^–/–^*) neutrophils compared with G1 (pDC^+^ lungs; *Stat1^+/+^*) neutrophils (Figure 4, B and D), consistent with prior experimental results (Figure 3E). Critically, pDC ablation increased the frequency of *Stat1^+/+^* neutrophils that contained live conidia (comparison of G2 versus G1 neutrophils; *P* = 0.027, Figure 4, B and D). This result indicates that pDC-derived products contribute to STAT1-dependent neutrophil conidiacidal activity. In contrast, pDC ablation did not significantly increase the frequency of *Stat1^–/–^* neutrophils that contained live conidia (comparison of G4 versus G3 neutrophils; *P* = 0.09, Figure 4, B and D).

To obtain a complementary measurement of pDC to neutrophil STAT1 crosstalk, we measured ROS production in the 4 neutrophil groups in parallel. Consistent with the direct measurements of fungicidal activity shown in Figure 4D and with the ROS measurements in Figure 3, I and J, *Stat1^+/+^* neutrophils displayed a higher ROS mean fluorescence intensity (MFI) than *Stat1^–/–^* neutrophils (G1 versus G3). pDC ablation markedly reduced ROS production by *Stat1^+/+^* neutrophils (G2 versus G1), in line with the reduction in neutrophil fungicidal activity (Figure 4E). In fact, *Stat1^+/+^* neutrophils isolated from pDC-depleted lungs (G2) had a similar ROS MFI as *Stat1^–/–^* neutrophils isolated from pDC-sufficient lungs (G3). ROS levels observed in *Stat1^–/–^* neutrophils isolated from pDC-depleted lungs (G3) were lower than the ROS levels observed in *Stat1^–/–^* neutrophils isolated from pDC-sufficient lungs (Figure 4E), consistent with the idea that pDCs can further modulate neutrophil ROS production independent of *Stat1* gene expression. Collectively, these findings indicate that pDCs regulate STAT1-dependent neutrophil fungicidal activity and ROS production.

### STAT1-dependent guanylate-binding proteins are dispensable for the neutrophil antifungal activity.

**Figure 5:**
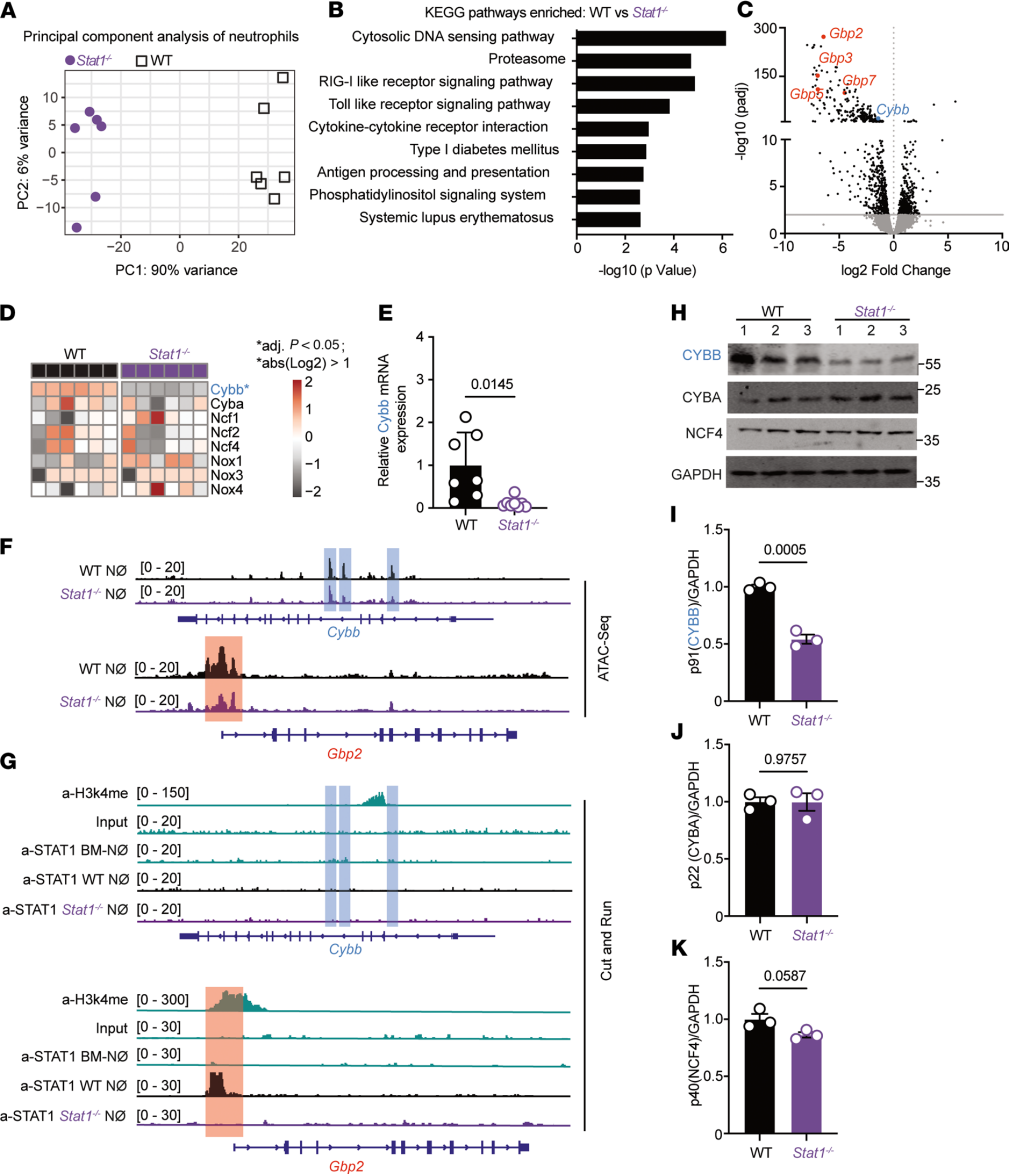
STAT1-dependent control of *Cybb* expression and CYBB protein levels in neutrophils. (**A**) Principal component analysis of global gene expression, (**B**) differentially enriched KEGG pathways, and (**C**) volcano plot of DEGs in *Stat1^–/–^* (purple symbols) and *Stat1^+/+^* (black symbols) neutrophils sorted from mixed BM chimeric mice (*n* = 6) at 72 hpi with 3 × 10^7^ CEA10 conidia. Selected downregulated genes in *Stat1^–/–^* neutrophils are highlighted in the volcano plot. (**D**) Heatmap of *Cybb*, *Cyba*, *Ncf1*, *Ncf2*, *Ncf4*, *Nox1*, *Nox3*, and *Nox4* expression in neutrophils sorted from *Stat1^–/–^* and *Stat1^+/+^* mixed BM chimeric mice (*n* = 6). The asterisk indicates that a gene is a DEG. Each lane represents an independent biological replicate. (**E**) qRT-PCR of *Cybb* mRNA expression in neutrophils sorted from *Stat1^–/–^* and *Stat1^+/+^* mice. (**F**) ATAC-Seq analysis of neutrophils sorted from *Stat1^–/–^* and *Stat^+/+^* mice. Gene tracks show chromatin accessibility at the *Cybb* and *Gbp2* locus. (**G**) BM and lung neutrophils were sorted from *Stat1^–/–^* and *Stat1^+/+^* mice and processed for CUT&RUN. Gene tracks show STAT1 signal as normalized fragment pileup (*y* axis) plotted by genome position (*x* axis). The shaded box highlights a putative STAT1 binding site at the *Gbp2* promoter region. Gene tracks show H3K4me3 ChIP-Seq signal as normalized fragment pileup (top rows; green). (**H**– **K**) Western blot (**H**) and quantitation of (**I**) CYBB, (**J**) CYBA, (**K**) NCF4 protein levels normalized to GAPDH expression in lung neutrophils sorted from *Stat1^–/–^* and *Stat1^+/+^* mice. Neutrophils from 4–5 mice were pooled to obtain sufficient protein for Western blot analysis in each biological replicate. **A**–**K**: Infection dose: 3 × 10^7^ CEA10 conidia via intratracheal route; analysis 72 hours p.i. **E**–**K**: Data are representative of 2 experiments. Data points represent individual mice. Statistical analysis: Mann-Whitney *U* test. See also Supplemental Figure 5.

To gain further insight into how STAT1 regulates cell-intrinsic neutrophil fungicidal activity and ROS generation during respiratory *A*. *fumigatus* challenge, we generated mixed BM chimeric mice (1:1 mix of CD45.2^+^ *Stat1*^−/−^ and CD45.1^+^ *Stat1^+/+^* donor BM cells → lethally irradiated CD45.1^+^CD45.2^+^ *Stat1*^+^^/+^ recipient mice) and performed bulk RNA-Seq on *Stat1*^−/−^ and *Stat1^+/+^* neutrophils sorted from *A*. *fumigatus*–infected recipient mice. There were marked differences in the transcriptomes of *Stat1*^−/−^ and *Stat1^+/+^* lung neutrophils (Figure 5A), with 2,586 genes showing differential expression in *Stat1*^−/−^ compared with *Stat1^+/+^* neutrophils. KEGG pathway enrichment analysis showed downregulation of many pathways in *Stat1*^−/−^ neutrophils, including the cytosolic DNA sensing, proteasome, RIG-I–like receptor signaling, Toll-like receptor signaling, and cytokine–cytokine receptor interaction pathways (Figure 5B). We identified commonly downregulated genes in *Stat1*^−/−^ neutrophils, many of which were known IFN-regulated genes (ISGs; Figure 5C).

Several GTPases, guanylate-binding proteins (GBPs), including *Gbp2*, *Gbp3*, *Gbp5*, and *Gbp7,* were significantly downregulated in *Stat1*^−/−^ neutrophils (Figure 5C). To address the function of these genes in neutrophil antifungal activity in otherwise immune-competent mice, we utilized Gbp^chr3−/−^ mice, which lack the entire chromosome 3 cluster that contains *Gbp1*, *Gbp2*, *Gbp3*, and *Gbp5*. We generated single chimeric mice (CD45.2^+^ Gbp^chr3−/−^ or CD45.2^+^ Gbp^chr3+/+^ → lethally irradiated CD45.1^+^ Gbp^chr3+/+^ recipients) (Supplemental Figure 5A) and compared the mortality and fungal burden of Gbp^chr3−/−^ and Gbp^chr3+/+^ chimeric mice. As expected based on a prior study in corticosteroid- and cyclophosphamide-treated mice (31), there was no difference in mortality or fungal CFU between Gbp^chr3−/−^ and Gbp^chr3+/+^ chimeric mice (Supplemental Figure 5, B and C). Using mixed chimeric mice (CD45.2^+^ Gbp^chr3−/−^ and CD45.1^+^ Gbp^chr3+/+^ → CD45.1^+^CD45.2^+^ Gbp^chr3+/+^ recipients) (Supplemental Figure 5D) and FLARE conidia, we quantified the cell-intrinsic antifungal activity of Gbp^chr3−/−^ and Gbp^chr3+/+^ leukocytes and found no difference in neutrophil conidial uptake and killing (Supplemental Figure 5, E and F). Moreover, Gbp^chr3−/−^ and Gbp^chr3+/+^ neutrophils isolated from the same lung exhibited no difference in ROS MFI (Supplemental Figure 5G).

### The pDC/IFN/STAT1 axis regulates neutrophil Cybb expression during A. fumigatus infection.

Since GBPs did not contribute to STAT1-regulated neutrophil defense against *A*. *fumigatus*, we investigated other candidate genes that were downregulated in *Stat1*^−/−^ neutrophils (Figure 5C) and focused on *Cybb,* which encodes CYBB, the p91 subunit of the NADPH oxidase complex (NOX2). The genes (*Cyba*, *Ncf1*, *Ncf2*, and *Ncf4*) that encode the 4 other NOX2 subunits (CYBA/p22 subunit, NCF1/p47 subunit, NCF2/p67 subunit, and NCF4/p40 subunit) were not downregulated in *Stat1^−/−^* neutrophils (Figure 5D). During *A*. *fumigatus* infection, whole-lung *Cybb* expression markedly increased at 48 and 72 hpi (Supplemental Figure 5H), temporally coincident with lung pDC influx and type I and type III IFN expression (Figure 2, A–C). NOX2-dependent ROS production is a key anti-*Aspergillus* defense mechanism, and we confirmed that *Cybb*-deficient mice were highly susceptible to *A*. *fumigatus* infection (Supplemental Figure 5, I–K) (32). To investigate the hypothesis that STAT1 signaling in neutrophils regulates *Cybb* expression, we isolated neutrophils from infected *Stat1*^−/−^ and *Stat1^+/+^* mice and found that *Cybb* mRNA level were decreased in *Stat1*^−/−^ neutrophils compared with *Stat1^+/+^* neutrophils (Figure 5E), consistent with STAT1-dependent regulation of *Cybb* transcription.

To determine chromatin accessibility at the *Cybb* locus, we performed assay for transposase-accessible chromatin (ATAC) sequencing of sorted *Stat1*^−/−^ and *Stat1^+/+^* lung neutrophils from *A*. *fumigatus*–infected mice. Within the *Cybb* locus, we found 3 regions (blue squares) that were less accessible in *Stat1*^−/−^ neutrophils than in *Stat1^+/+^* neutrophils (Figure 5F), consistent with a modest STAT1-dependent regulation of *Cybb* expression (Figure 5, D and E). As a positive control, within the Gbp2 locus, we found 1 region (red square) that was less accessible in *Stat1*^−/−^ neutrophils compared with *Stat1^+/+^* neutrophils (Figure 5F), consistent with STAT1-dependent regulation of *Gbp* expression (Figure 5C).

To probe STAT1 binding to the *Cybb* gene locus, we performed cleavage under targets and release using nuclease (CUT&RUN) experiments of sorted *Stat1*^−/−^ and *Stat1^+/+^* lung neutrophils from *A*. *fumigatus*–infected mice. We did not observe direct STAT1 binding to the *Cybb* locus and promoter region (Figure 5G). In CUT&RUN experiments, STAT1 bound to the *Gbp2* locus and promoter region (Figure 5G). Collectively, these results suggest that STAT1 regulates *Cybb* transcription via an indirect mechanism.

We next explored whether lung neutrophil CYBB protein levels were regulated by STAT1 signaling. *Stat1*^−/−^ and *Stat1^+/+^* neutrophils were isolated from *A*. *fumigatus*–infected lungs at 72 hpi and analyzed for CYBB expression by Western blotting. Protein levels of CYBB, but not of CYBA and NCF4, were lower in *Stat1*^−/−^ neutrophils than in *Stat1^+/+^* neutrophils (Figure 5, H–K), linking neutrophil STAT1 activation to CYBB expression and to the oxidative burst.

## Discussion

Our data introduce a model of protective pDC-neutrophil crosstalk in which pDCs undergo a defined activation trajectory in transit to the *Aspergillus*-infected lung. pDC activation provides a critical source of type I and type III IFN at the portal of infection and licenses neutrophil antifungal properties in the lung in a direct manner. The latter step occurs through neutrophil-intrinsic STAT1-dependent control of CYBB protein levels. Local pDC-dependent control of NADPH oxidase assembly regulates the strength of neutrophil oxidative burst, as judged by ROS production; boosts neutrophil intracellular conidial killing; and confers sterilizing immunity against inhaled spores.

pDCs originate in the BM, travel through the bloodstream, and migrate to lymphoid and nonlymphoid tissues during both normal and inflammatory states (18, 33–35). In a recent study (18), we demonstrated that *Aspergillus*-infected Mo-DCs and neutrophils release CXCR3 ligands (i.e., CXCL9 and CXCL10) into the inflamed lung, coupling fungal recognition and fungus-induced inflammation to CXCR3 signaling–dependent pDC influx from the circulation into the lung. In the present study, *A*. *fumigatus* infection induces a pDC activation trajectory that substantially alters the lung rather than the BM pDC transcriptome, implying that pDC trafficking to peripheral tissue precedes activation occurs at the portal of infection. While not a focus of this work, the precise mechanism of pDC activation in the fungus-infected lung remains an open question, in part because conditional gene deletion strategies do not exist for pDCs, precluding facile comparison of gene-deficient and gene-sufficient pDCs in infected tissues in mice with no other genetic perturbations.

Our data indicate that lung-infiltrating pDCs upregulate genes implicated in fungal recognition, including C-type lectin receptors (CLRs) and downstream signaling molecules. pDCs express the C-type lectin receptors dectin-1, dectin-2, and dectin-3 and can secrete IFN-α and TNF when coincubated with *Aspergillus* hyphae via dectin-2 signaling (36–38), though the interaction of pDCs with conidia, the infectious propagules, was not examined. pDCs do not internalize *Aspergillus* conidia readily in the test tube or in the infected lung (4, 36), supporting the notion that activation occurs via fungal cell contact or via the presence of activating cytokines or other inflammatory mediators. Support for the latter scenario comes from an experiment in which upregulation of pDC CD40 and CD83 expression induced by curdlan (i.e., a particulate dectin-1 agonist) could be further increased by simultaneous coincubation with an acellular curdlan-stimulated peripheral blood mononuclear cell supernatant (36). Beyond *Aspergillus*, pDC-enriched human cell fractions can release TNF in a dectin-2 and dectin-3 signaling–dependent fashion in response to *Paracoccidioides brasiliensis* coincubation (39). Beyond CLR signaling, we found that *Aspergillus* infection triggered upregulation of Toll-like receptors in lung-infiltrating pDCs. Prior work demonstrated that *Aspergillus*-derived unmethylated CpG sequences can activate TLR9 signaling in vitro (40, 41), and this may represent an additional mechanism by which pDCs become activated during *Aspergillus* infection.

During Dengue, Zika, and hepatitis C viral infections, physical contact with virally infected cells stimulates pDC-mediated antiviral responses (42, 43). α_L_β_2_ integrin (lymphocyte function–associated antigen-1 [LFA1]) expressed by the pDC can bind to ICAM1 on infected cells to promote a sustained interaction — termed an interferonogenic synapse — during which viral RNA is transferred to pDCs, leading to IFN production via the nucleic acid sensor TLR7. This process activates type I IFN–dependent antiviral programs in infected tissues. pDC commitment to type I IFN production is further regulated by antecedent cell-intrinsic TNF receptor and leukemia inhibitory factor signaling during murine cytomegalovirus infection, underscoring the contribution of local cytokine signaling to pDC activation by contact-dependent and -independent mechanisms (44). The lung pDC transcriptomic data indicated increased expressed ICAM1 in the lung-infiltrating pDCs isolated from *Aspergillus*-infected mice. This observation raises the possibility that ICAM1 expression may facilitate lung pDC contact with β_2_-integrin–expressing myeloid cells to facilitate reciprocal interactions. In sum, the upregulation of multiple receptors from various classes suggests that pDCs likely employ a blend of receptors to identify and react to fungal pathogens or host cells, leading to full pDC activation in the fungus-infected lung. Notably, we were unable to isolate sufficient pDCs to compare the bulk lung pDC transcriptome in naive mice with the lung pDC transcriptome in *Aspergillus*-infected mice.

Recent studies have advanced the concept that circulating neutrophils exhibit transcriptional heterogeneity in the steady state and during microbial infection (45). In addition, neutrophils can acquire transcription-dependent noncanonical functions upon entry into peripheral tissues, exemplified by a regulatory role in angiogenesis in the lung (46). Single-cell analysis has revealed the presence of 3 separate circulating murine and human populations that differ with respect to IFN-stimulated gene (ISG) and CXCR4 expression. Interestingly, the 3 populations expressed similar levels of *Cybb* mRNA and exhibited similar NADPH oxidase scores by gene ontogeny analysis during homeostasis and systemic bacterial (i.e., *Escherichia coli*) infection (45). During tuberculosis, malaria, and hematopoietic cell transplantation, circulating human neutrophils exhibit an IFN transcriptional signature (47–49), which in the case of malaria, is reduced by receipt of antimalarial therapy. The finding that pDC-derived type I and type III IFN licenses neutrophil antifungal activity through STAT1-dependent control of *Cybb* transcription and CYBB protein levels raises the important question of whether the ISG^hi^ neutrophil subset is enriched in the lung and particularly effective at killing *Aspergillus* conidia compared with the other 2 ISG^lo^ circulating subsets. The ability to track and quantify fungal uptake killing with FLARE conidia should facilitate future studies to link lung-infiltrating neutrophil subsets, defined by distinct transcriptional profiles, to the quality of effector functions. These studies support the idea that dynamic transcriptional plasticity represents a cardinal feature of the circulating neutrophil response to microbial infection and other external stressors. It remains unclear whether the observed transcriptional plasticity represents dynamic changes in the production of transcriptionally heterogeneous circulating neutrophil subsets or represents an interconversion between circulating subsets.

Our findings support a model in which pDC-derived IFNs enhance neutrophil-mediated conidial killing in the lung. A limitation the murine infection model is that FLARE conidia do permit quantitation of neutrophil-mediated antihyphal activity in vivo. Due to the absence of an in vivo readout for hyphal killing, we were unable to directly compare this function in pDC-sufficient and pDC-depleted mice. Regarding extracellular hyphal killing, prior work demonstrated that *Cybb^–/–^* thioglycolate-elicited neutrophils exhibit impaired hyphal killing in vitro compared with WT controls (50).

The effects of type III IFN on neutrophils are context dependent. In a dextran sodium sulfate (DSS) model of colitis, IFN-λ has been linked to neutrophil ROS suppression and a reduction in neutrophil degranulation (51). In human neutrophils, recombinant IFN-λ can inhibit platelet-induced NETosis (52). IFN-λ has various roles in bacterial infections (53, 54); a study demonstrated that *Bordetella pertussis* infection induces IFN-λ and IFN-λ receptor (IFNLR1) expression as well as inflammation in the lung (53). IFN-λ signaling in neutrophils suppressed *B*. *pertussis* killing and neutrophil production of ROS, MMP9, NETs, MPO, and IFN-γ (55, 56). In contrast, conditional deletion of the IFN-λ receptor in neutrophils was linked to a reduction in neutrophil ROS during pulmonary *A*. *fumigatus* infection (15). During HSV corneal infection, application of recombinant IFN-λ suppressed neutrophil recruitment but did not impact virucidal activity or ROS production (57). These results indicate that IFN-λ may have different biological effects based on cellular targets and responsiveness at sites of inflammation and on the type of inflammatory stimulus. In this study, the STAT1-dependent action of pDC-dependent IFNs (both type I and III) on neutrophils was critical for local licensing of their cell-intrinsic cytotoxic activity against *Aspergillus* conidia. The individual contribution of type I versus type III IFN–dependent activation on STAT1-dependent CYBB expression remains unclear. An open question remains how STAT1 signaling precisely regulates *Cybb* transcription and CYBB translation, since we could not detect clear evidence of STAT1 binding to the *Cybb* promoter or marked STAT1-dependent changes in chromatin accessibility.

Overall, these findings provide insights into the pDC/STAT1/CYBB axis as a key regulator of NADPH oxidase expression and highlight the critical role of this pathway in promoting antifungal immunity in neutrophils. The discovery that pDCs regulated ROS induction by neutrophils by controlling the STAT1-dependent expression of a single NADPH oxidase subunit adds to our understanding of the complex and protective interplay between innate immune cells during fungal infection.

## Methods

### Sex as a biological variable

Previous studies in our laboratories have not identified sex-specific differences in murine susceptibility to Aspergillus fumigatus. In our experiments, we used both female and male mice aged 8–12 weeks. For each study, sex was considered a biological variable, and the sex of each mouse was recorded to assess potential sex-related differences in outcomes. Data were analyzed with respect to sex, and results were pooled from experiments in which male and female mice were equally distributed across experimental and control groups.

### Mice

C57BL/6J (JAX: 00664), BDCA2-DTR (JAX: 014176) mice were from Jackson Laboratories. *GBP^chr3–/–^* BMs were provided by Thirumala-Devi Kanneganti (St. Jude Children’s Research Hospital, Memphis, Tennessee). C57BL/6.SJL mice (Stock: 4007) were purchased from Taconic. C57BL/6 and C57BL/6.SJL mice were crossed to generate CD45.1^+^CD45.2^+^ recipient mice for mixed BM chimeras. CD45.2^+^ BDCA2-DTR^Tg/+^ were backcrossed to C57BL/6.SJL mice to obtain CD45.1^+^ BDCA2- DTR^Tg/+^ mice. BDCA2- DTR^Tg/+^ were crossed with CD45. 2^+^ *Stat1^–/–^* mice to obtain CD45. 2^+^ BDCA2- DTR^Tg/+^ *Stat1^–/–^* mice. For experiments in which the breeding strategy did not yield littermate controls, gene-knockout mice were co-housed with C57BL/6 mice for 14 days prior to infection, whenever possible.

### Generation of BM chimeric mice

For single BM chimeras, CD45.1^+^ C57BL/6.SJL recipients were lethally irradiated (900cGy), reconstituted with either 2–5 × 10^6^ CD45.2^+^ *GBP^chr3–/–^*, CD45.2^+^ C57BL/6J or CD45.2^+^ *Stat1^–/–^* BM cells. For mixed BM chimeras, CD45.1^+^CD45.2^+^ recipients were irradiated and reconstituted with a 1:1 mixture of CD45.1^+^ C57BL/6.SJL and CD45.2^+^ *Stat1^–/–^* or CD45.2^+^ *GBP^chr3–/–^* BM cells, CD45.1^+^ BDCA2- DTR^Tg/+^ and CD45.2^+^ BDCA2- DTR^Tg/+^ *Stat1^–/–^*. After BM transplantation, recipient mice received 400 g/mL enrofloxacin in the drinking water for 21 days to prevent bacterial infections and rested for 6-8 weeks prior to experimental use.

### Chemicals and reagents

Unless otherwise noted, chemicals were purchased from Sigma-Aldrich or Fisher Scientific, cell culture reagents from Thermo Fisher Scientific, and microbiological culture media from BD Biosciences. Antibodies for flow cytometry were acquired from BD Biosciences, Thermo Fisher Scientific and Tonbo. Comprehensive details of all reagents are provided in Supplemental Tables 1–5.

### Aspergillus fumigatus infection model

*A. fumigatus* Af293, Af293-dsRed (25), and CEA10 (58) strains were cultured on glucose minimal medium slants at 37°C for 4–7 days prior to harvesting conidia for experimental use. To generate FLARE conidia, briefly, 7 × 10^8^ Af293-dsRed conidia were rotated in 10 μg/mL Biotin XX, SSE in 1 mL of 50 mM carbonate buffer (pH 8.3) for 2 hours at 4°C, incubated with 20 μg/mL Alexa Fluor 633 succinimidyl ester at 37°C for 1 hour, resuspended in PBS and 0.025% Tween 20 for use within 24 hours. (25, 59). To generate morphologically uniform heat-killed swollen conidia, 5 × 10^6^/mL conidia were incubated at 37°C for 14 hours in RPMI-1640 and 0.5 μg/mL voriconazole and heat killed at 100°C for 30 minutes (60). To infect mice with 30–60 million live or heat-killed *A*. *fumigatus* cells, conidia were resuspended in PBS, 0.025% Tween-20 at a concentration of 0.6–1.2 × 10^9^ cells and 50 μL of cell suspension was administered via the intranasal route to mice anesthetized by isoflurane inhalation.

### Analysis of in vivo and in vitro conidial uptake and killing

To analyze of conidia uptake and killing, FLARE conidia were used to infect mice. In data analyses for a given leukocyte subset, conidial uptake refers to the frequency of fungus-engaged leukocytes, i.e., the sum of dsRed^+^ AF633^+^ and dsRed^–^ AF633^+^ leukocytes. Conidial viability within a specific leukocyte subset refers to the frequency of leukocytes that contain live conidia (dsRed^+^ AF633^+^) divided by the frequency of all fungus-engaged leukocytes (dsRed^+^ AF633^+^ and dsRed^–^ AF633^+^).

### In vivo cell depletion

To ablate specific cells, BDCA2- DTR^Tg/+^, BDCA2- DTR^Tg/+^ *Stat1^–/–^*, and nontransgenic littermate controls were injected i.p. with 10 ng/g body weight DT on Day -1, Day 0, and Day +2 p.i. (19), unless noted otherwise.

### Analysis of infected mice

To prepare single cell lung suspensions for flow cytometry, we followed the method outlined in (61) with minor modifications. After perfusing murine lungs, they were placed in a gentle MACS C tube and mechanically homogenized in 5 mL RPMI-1640, 10% FBS, and 0.1 mg/mL DNAse using a gentle MACS Octo Dissociator (Miltenyi Biotech) without the use of collagenase. The lung cell suspensions were then lysed of RBCs, enumerated, and stained with fluorophore-conjugated antibodies prior to flow cytometric analysis. We used either a Cytoflex or a BD Aria for flow cytometric sorting and performed flow plot analysis using FlowJo v.9.6.6 software.

Neutrophils were identified as CD45^+^CD11b^+^Ly6C^lo^Ly6G^+^ cells, monocytes as CD45^+^CD11b^+^CD11c^–^Ly6G^–^Ly6C^hi^ cells, Mo-DCs as CD45^+^CD11b^+^CD11c^+^Ly6G^–^Ly6C^hi^ MHC class II^+^ cells, and pDCs as CD45^+^CD11c^int^SiglecF- CD19^–^NK1.1^–^CD11b^–^B220^+^ SiglecH^+^ cells.

To assess the lung fungal burden, perfused murine lungs were homogenized using a PowerGen 125 homogenizer (Fisher) in 2 mL of PBS containing 0.025% Tween-20 and plated onto Sabouraud dextrose agar. For analysis of cytokine levels by ELISA, whole lungs were weighed and mechanically homogenized in 2 mL of PBS containing protease inhibitors. To analyze cytokine levels by qRT-PCR, we extracted total RNA from cells using TRIzol (Invitrogen). One to 2 micrograms of total RNA were reverse-transcribed using the High-Capacity cDNA Reverse Transcription Kit (Applied Biosystems). We used TaqMan Fast Universal Master Mix (2×) and TaqMan probes (Applied Biosystems) for each gene and normalized to glyceraldehyde-3-phosphate dehydrogenase. Gene expression was calculated using the Ct method relative to the naive sample.

Intracellular ROS levels were measured in cells using CM-H2DCFDA [5-(and 6-) chloromethyl-2,7-dichlorodihydrofluorescein diacetate, acetyl ester] as described in (62). Briefly, single cell lung suspensions were incubated with 1 μM CM-H2DCFDA in Hanks’ balanced salt solution at 37°C for 45 minutes according to manufacturer’s instruction and analyzed by flow cytometry.

### Immunoblotting assay

Cells were lysed in lysis buffer (150 mM NaCl, 50 mM HEPES (pH 7.4), 1 mM EDTA, 1% Nonidet P-40, protease inhibitors). Total cell lysates were subjected to SDS-PAGE and then blotted using indicated antibodies, Cybb polyclonal antibody (Invitrogen, cat. no. PA5-76034), p22phox mAb (CST, cat. no. 37570), p40phox mAb (cat. no. AB76158), GAPDH mAb (CST, cat. no. 5174s).

### RNA sequencing

#### RNA extraction.

Phase separation in cells lysed in 1 mL TRIzol Reagent (ThermoFisher cat. no. 15596018) was induced with 200 μL chloroform. RNA was extracted from 350 μL of the aqueous phase using the miRNeasy Micro or Mini Kit (Qiagen cat. no. 217084/217004) on the QIAcube Connect (Qiagen) according to the manufacturer’s protocol. Samples were eluted in 13-15 μL RNase-free water.

#### Transcriptome sequencing.

After RiboGreen quantification and quality control by Agilent BioAnalyzer, 1.9-2.0 ng total RNA with RNA integrity numbers ranging from 7.8 to 10 underwent amplification using the SMART-Seq v4 Ultra Low Input RNA Kit (Clonetech cat. no. 63488), with 12 cycles of amplification. Subsequently, 7.4–10 ng of amplified cDNA was used to prepare libraries with the KAPA Hyper Prep Kit (Kapa Biosystems cat. no. KK8504) using 8 cycles of PCR. Samples were barcoded and run on a HiSeq 4000 or NovaSeq 6000 in a PE50 (HiSeq) or PE100 (NovaSeq) run, using the HiSeq 3000/4000 SBS Kit or NovaSeq 6000 S4 Reagent Kit (200 Cycles) (Illumina). An average of 40 million paired reads were generated per sample and the percent of mRNA bases per sample averaged 84%.

### RNA-Seq data analysis

Raw reads were quality checked with FastQC v0.11.7 (http://www.bioinformatics.babraham.ac.uk/projects/fastqc/), and adapters were trimmed using Trim Galore v0.6.7 (http://www.bioinformatics.babraham.ac.uk/projects/trim_ galore/). Reads were aligned to the mouse reference genome (GRCm38.p6) using STAR v2.6.0c (63) with default parameters. Gene abundances were calculated with featureCounts v1.6.2 (64) using composite gene models from Gencode release vM17. Principal component analysis was performed using the plotPCA function from DESeq2 v1.32.0 (65). DEGs were determined with DESeq2 v1.32.0 with a 2-factor model incorporating batch as a covariate, with significance determined by Wald tests (*q* < 0.05). Gene set enrichment analysis was performed using fgsea v1.18.0 (66) with gene sets from the Broad Institute’s MSigDB (67, 68) collections; genes were ranked by the DESeq2 Wald statistic. Only pathways with an adjusted *P* value < 0.05 were considered enriched. Expression heatmaps were generated using variance-stabilized data, with the values centered and scaled by row.

### RNAscope microscopy

Formaldehyde-fixed, paraffin embedded (FFPE) surgical tissue Sects. FFPE tissue sections (5 μm) were processed exactly as described in the manufacturer’s instructions (ACD Bio, cat. no. 323100). Probes targeting the following genes were used: Ly6G (ACD Bio, cat. no. 455701-C3), IFNλR (ACD Bio, cat. no. 512981-C1), IFN*a*R1(ACD Bio, cat. no. 512971-C2). Slides were mounted with Prolong Diamond mounting media (ThermoFisher Scientific, cat. no. P36965). Slides were scanned using a Pannoramic Digital Slide 517 Scanner (3DHISTECH, Budapest, Hungary) using a 20/0.8NA objective.

### ATAC sequencing

Freshly harvested WT and KO mouse neutrophils were directly sent to MSKCC’s Epigenetics Research Innovation Lab. ATAC was performed as previously described (Corces et al. Nature Methods 2017 (69) using 50,000 cells per replicate and the Tagment DNA TDE1 Enzyme (Illumina, 20034198). Sequencing libraries were prepared using the ThruPLEX DNA-Seq Kit (Takarabio, R400676) and sent to the MSKCC Integrated Genomics Operation core facility for sequencing on a NovaSeq 6000. Raw sequencing reads were trimmed and filtered for quality (Q>15) and adapter content using version 0.4.5 of TrimGalore (https://www.bioinformatics.babraham.ac.uk/projects/trim_galore) and running version 1.15 of cutadapt and version 0.11.5 of FastQC. Version 2.3.4.1 of bowtie2 (http://bowtie-bio.sourceforge.net/bowtie2/index.shtml) was employed to align reads to mouse assembly mm10 and alignments were deduplicated using Mark Duplicates in Picard Tools v2.16.0. Enriched regions were discovered using MACS2 (https://github.com/taoliu/MACS) with a *P* value setting of 0.001, filtered for blacklisted regions (http://mitra.stanford.edu/kundaje/akundaje/release/blacklists/ mm10-mouse/mm10.blacklist.bed.gz), and a peak atlas was created using +/- 250 bp around peak summits. The BEDTools suite (http://bedtools.readthedocs.io) was used to create normalized bigwig files. Version 1.6.1 of featureCounts (http://subread.sourceforge.net) was used to build a raw counts matrix and DESeq2 was employed to calculate differential enrichment for all pairwise contrasts. Peak-gene associations were created by assigning all intragenic peaks to that gene, while intergenic peaks were assigned using linear genomic distance to transcription start sites (TSS).

### Histone and transcription factor CUT&RUN analyses

For CUT&RUN, 400,000 sorted neutrophils were used for H3K4me3 and STAT1 analysis. Anti-H3K4me3 (Epicypher, cat. no. 13-0028) or polyclonal anti-STAT1 antibodies (Proteintech, cat. no. 10144-2-AP) were employed for each target. Sorted cells were washed with PBS and resuspended in Antibody Buffer (1 eBioscience Perm/Wash Buffer, 1 Roche cOmplete EDTA-free Protease Inhibitor, 0.5 μM Spermidine, and 2 μM EDTA in H2O). They were incubated overnight at 4°C with control IgG, H3K4me3, or STAT1 antibodies diluted 1:100 in Antibody Buffer in a 96-well V-bottom plate. Following antibody incubation, cells were washed twice with Buffer 1 (1 eBioscience Perm/Wash Buffer, 1 Roche cOmplete EDTA-free Protease Inhibitor, 0.5 μM Spermidine in H2O) and resuspended in 50 μL of Buffer 1 plus 1 pA/G-MNase (Cell Signaling, cat. no. 57813). This mixture was incubated on ice for 1 hour, then washed twice with Buffer 2 (0.05% w/v Saponin, 1 Roche cOmplete EDTA-free Protease Inhibitor, 0.5 μM Spermidine in 1X PBS) 3 times. Cells were resuspended in Calcium Buffer (Buffer 2 plus 2 μM CaCl2) and incubated on ice for 30 minutes to activate the pA/G-MNase reaction. An equal volume of 2 STOP Buffer (Buffer 2 plus 20 μM EDTA plus 4 μM EGTA) and 1 pg of *Saccharomyces cerevisiae* spike-in DNA (Cell Signaling, cat. no. 29987) were added. Samples were incubated at 37°C for 15 minutes, followed by DNA isolation and purification using the Qiagen MinElute Kit per the manufacturer’s instructions.

Immunoprecipitated DNA was quantified by PicoGreen, and the size was evaluated using an Agilent BioAnalyzer: fragments between 100 and 600 bp were size selected using aMPure XP beads (Beckman Coulter, cat. no. A63882). KAPA HTP Library Preparation Kit (Kapa Biosystems, cat. no. KK8234) was used to prepare Illumina sequencing libraries according to the manufacturer’s instructions with 0.001–0.5 ng input DNA and 14 cycles of PCR. Barcoded libraries were run on the NovaSeq 6000 in a PE100 run using S4 kit version 1.5 with XP mode to generate approximately 23 million paired reads per sample.

CUT&RUN datasets were processed by trimming paired reads for adaptors and low-quality sequences using Trimmomatic (v0.39) and aligning to the mm10 reference genome with Bowtie 2 (v2.4.1). Peaks were identified using MACS2 (v2.2.7.1) with input samples as control, employing narrow peak parameters and cutoff - analysis - ple-5 - keep - dup all -B - SPMR. Irreproducible discovery rate (IDR) calculations were performed using ENCODE project scripts (IDR v2.0.4.2). Reproducible peaks with an IDR value of 0.05 or less in each condition were retained, aggregated, and merged to create the final atlas, which was annotated with the UCSC Known Gene model. Reads were mapped to this atlas and counted using the summarize Overlaps function from the Genomic Alignment package (v1.34.1). Genomic tracks were visualized using GViz (v1.42.1) or IGV (v2.9.4).

### Statistics

All data presented are representative of at least 2 independent experiments, as indicated. Unless stated otherwise, all results are expressed as mean (± SEM). We used the Mann-Whitney *U* test for comparisons of 2 groups, and the Kruskal-Wallis test for multigroup comparisons, unless noted otherwise. Survival data were analyzed using the long-rank test. A *P* value less than 0.05 was considered significant; A *P* value greater than 0.05 was regarded as not statistically significant. All statistical analyses were performed using GraphPad Prism software (v9.2.0).

### Study approval

All mouse strains were bred and housed in the MSKCC or St. Jude Children’s Research Hospital Animal Resource Center under specific pathogen-free conditions. All animal experiments were conducted with sex- and age-matched mice and performed with MSKCC Institutional Animal Care and Use Committee approval. Animal studies complied with all applicable provisions established by the Animal Welfare Act and the Public Health Services Policy on the Humane Care and Use of Laboratory Animals.

### Data availability

Values for all data points in graphs are reported in the Supporting Data Values file. RNA-Seq data, ATAC-Seq data and CUT&RUN sequencing data were uploaded to the NCBI database. Raw sequencing datasets generated in this study were deposited in the NCBI’s Gene Expression Omnibus database (GEO GSE303829); raw RNA-Seq datasets, GSE280164; raw ATAC-Seq data, GSE303828; CUT&RUN sequencing data, GSE280229. RNA-Seq code has been deposited to GitHub (https://github.com/abcwcm/Guo2024; commit ID b6d43fb).

Author contributions

Conceptualization, TMH, and YG; methodology, TMH and YG; investigation, YG, MAA, KAMM, SAG, HK, PZ, MG, AB, KBM, YY, LH, and LCET; writing — original draft, YG and TMH; writing — review and editing, YG and TMH; funding acquisition, TMH and YG; resources, JCS, DB, and AR.

## Supplementary Material

Supplemental data

Unedited blot and gel images

Supporting data values

## Version 1 08/05/2025

In-Press Preview

## Version 2 10/15/2025

Electronic publication
