# Supplementary material for: An IFN/STAT1/CYBB axis defines protective plasmacytoid DC–neutrophil crosstalk in Aspergillus fumigatus–infected mice
Source: J Clin Invest. 2025 Aug 5;135(20):e190107. doi: 10.1172/JCI190107 (PMC12520677; doi:10.1172/JCI190107)
Supplement: Supplemental data [file jci-135-190107-s233.pdf]

An IFN/STAT1/CYBB Axis Defines Protective Plasmacytoid DC to Neutrophil Crosstalk in *Aspergillus fumigatus*-Infected mice

Yahui Guo[1, 2], Mariano A. Aufiero[1, 2, 3], Kathleen A.M. Mills[1, 2, 4], Simon A. Grassmann[5], Hyunu Kim[3, 5], Mergim Gjonbalaj[4], Paul Zumbo[6], Audrey Billips[1, 2], Katrina B. Mar[1, 2], Yao Yu[1, 2], Laura Echeverri Tirado[7], Lena Heung[8], Amariliz Rivera[7], Doron Betel[9], Joseph C. Sun[5], and Tobias M. Hohl[1, 2, 3, 4, 5, #]

[1]Infectious Disease Service, Department of Medicine, and [2]Human Oncology and Pathogenesis Program, [3]Louis V. Gerstner Jr. Graduate School of Biomedical Sciences, Sloan Kettering Institute, Memorial Sloan Kettering Cancer Center, New York, NY, USA

[4]Immunology and Microbial Pathogenesis Graduate Program, Weill Cornell Graduate School, New York, NY, USA

[5]Immunology Program, Sloan Kettering Institute, Memorial Sloan Kettering Cancer Center, New York, NY, USA

[6]Applied Bioinformatics Core, Department of Physiology and Biophysics, Weill Cornell Medicine, New York, NY, USA.

[7]Center for Immunity and Inflammation, New Jersey Medical School, Rutgers- The State University of New Jersey, Newark, New Jersey, USA.

[8]Department of Medicine and Department of Biomedical Sciences, Women's Guild Lung Institute, Cedars-Sinai Medical Center, Los Angeles, CA, USA.

[9]Applied Bioinformatics Core, Division of Hematology and Medical Oncology, Department of Medicine, Institute for Computational Biomedicine, Weill Cornell Medicine, New York, NY, USA.

[#], Corresponding author and lead contact. E-mail: hohlt@mskcc.org (T.M.H.)

Footnote

[#]Address correspondence to:

Tobias M. Hohl, MD, PhD

Memorial Sloan Kettering Cancer Center

1275 York Avenue, Box 9

New York, NY 10065

Phone: 646-888-2009

hohlt@mskcc.org

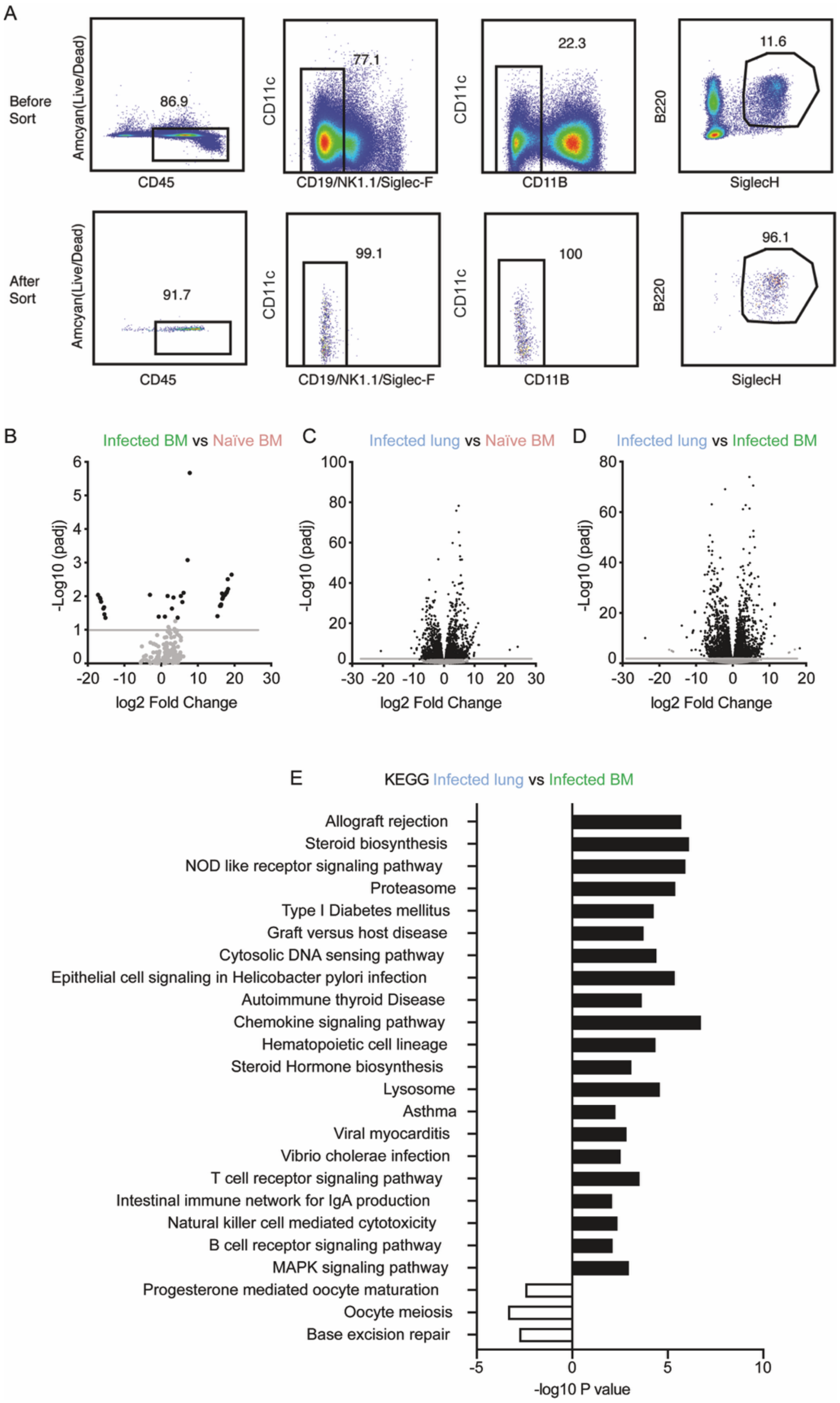

Figure S1. Related to Fig. 1. pDC transcriptome analysis following *A. fumigatus* infection.

(A) The plots indicate the FACS sorting strategy for BM and lung pDCs (top row) and the typical (>95%) pDC purity after FACS sorting (bottom row). (B) Volcano plot of the differentially expressed genes in pDCs sorted from the bone marrow of infected vs. naïve mice. (C) Volcano plot of the differentially expressed genes in pDCs sorted from infected lungs vs naïve bone marrow. (D) Volcano plot of the differentially expressed genes in pDCs sorted from infected lungs vs infected bone marrow. (E) The plot shows differentially exriched KEGG pathways ($q < 0.05$) observed in pDCs isolated from infected lungs vs infected BM. Black bars indicate pathways enriched in lung pDCs from infected mice, white bars indicate pathways enriched in BM pDCs from infected mice.

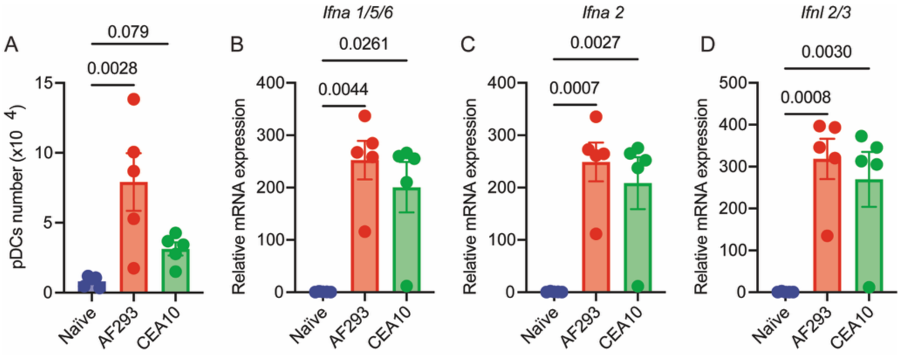

Figure S2. Related to Fig. 2. Fungal strain influence on pDCs recruitment and IFNs production.

(A) Lung pDC numbers in wildtype C57BL6/J mice at naïve status (blue symbol), and at 72h pi infected with 3×10^7 Af293 conidia (red symbols) and CEA10 conidia (green symbols), n = 5 per group. (B - D) *Ifn* genes expression, measured by qRT-PCR using TaqMan probes, in the lung of wildtype C57BL6/J mice at naïve status (blue symbol), and at 72h pi infected with 3×10^7 Af293 conidia (red symbols) and CEA10 conidia (green symbols), n = 5 per group. Statistical analysis: Kruskal-Wallis test.

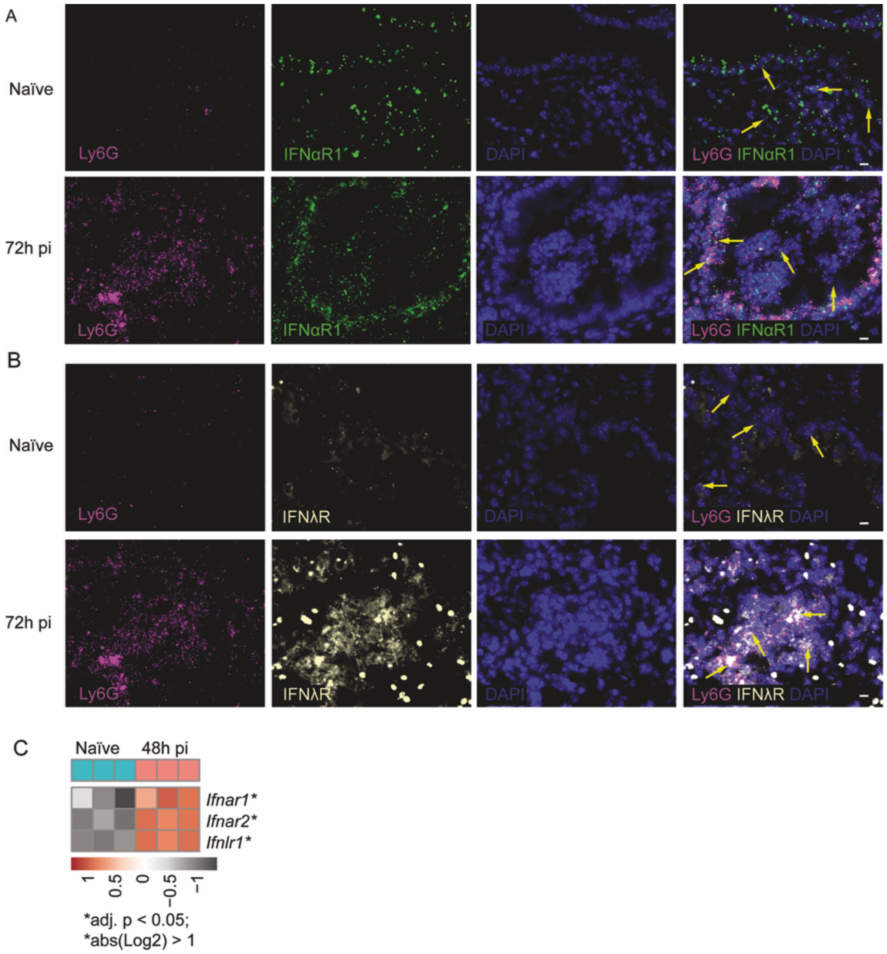

Figure S3. Related to Fig. 3. Type I and III IFN receptor expression on Ly6G⁺ lung neutrophils.

(A) Lung sections from naïve and *A. fumigatus*-infected mice were analyzed by RNAscope using probes to Ly6G (first column) and IFNαR1 (second column), and DAPI (third column), and merged images are shown in fourth column, and yellow arrows indicate examples of co-localization of the Ly6G and IFNαR1 probes within the same nuclei. (B) Lung sections from naïve and *A. fumigatus*-infected mice were analyzed by RNAscope using probes to Ly6G (first column) and IFNλR1 (second column), and DAPI (third column), and merged images are shown in fourth column, and yellow arrows

indicate examples of co-localization of the Ly6G and IFNλR1 probes within the same nuclei. (C) Differential gene expression as assessed by RNA-seq of pulmonary neutrophils isolated from uninfected controls (naïve) or mice infected with *A.fumigatus* CEA10 for 48 hours. Heat map depicts the 3 IFN receptors genes expressed at FC > 2.5 in *A. fumigatus* infected neutrophils compared to naïve neutrophils. (A and B) Infection dose: 3×10^7 CEA10 conidia via intratracheal route, analysis at 72 hpi. Scale bar = 20 μm.

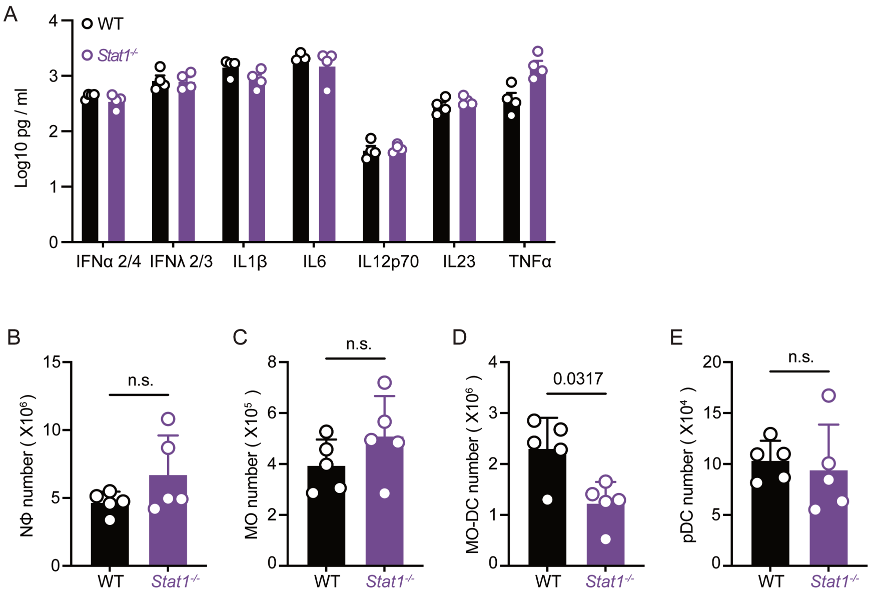

Figure S4. Related to Fig. 3. STAT1 modulates the neutrophil antifungal response. (A) Lung cytokine levels measured by ELISA of *Stat1*$^{-/-}$ (purple symbols) and *Statt1*$^{+/+}$ (black symbols) mice. (B - E) Lung (B) neutrophil, (C) monocyte, (D) Mo-DC and (E) pDC numbers in *Stat1*$^{-/-}$ (purple symbols) and *Stat1*$^{+/+}$ (black symbols). (A - E) Infection dose: 3 × 10^7 CEA10 conidia via intratracheal route, analysis 72 hpi. Data are representative of 2 experiments. Dots represent individual mice. Statistical analysis: Mann-Whitney test.

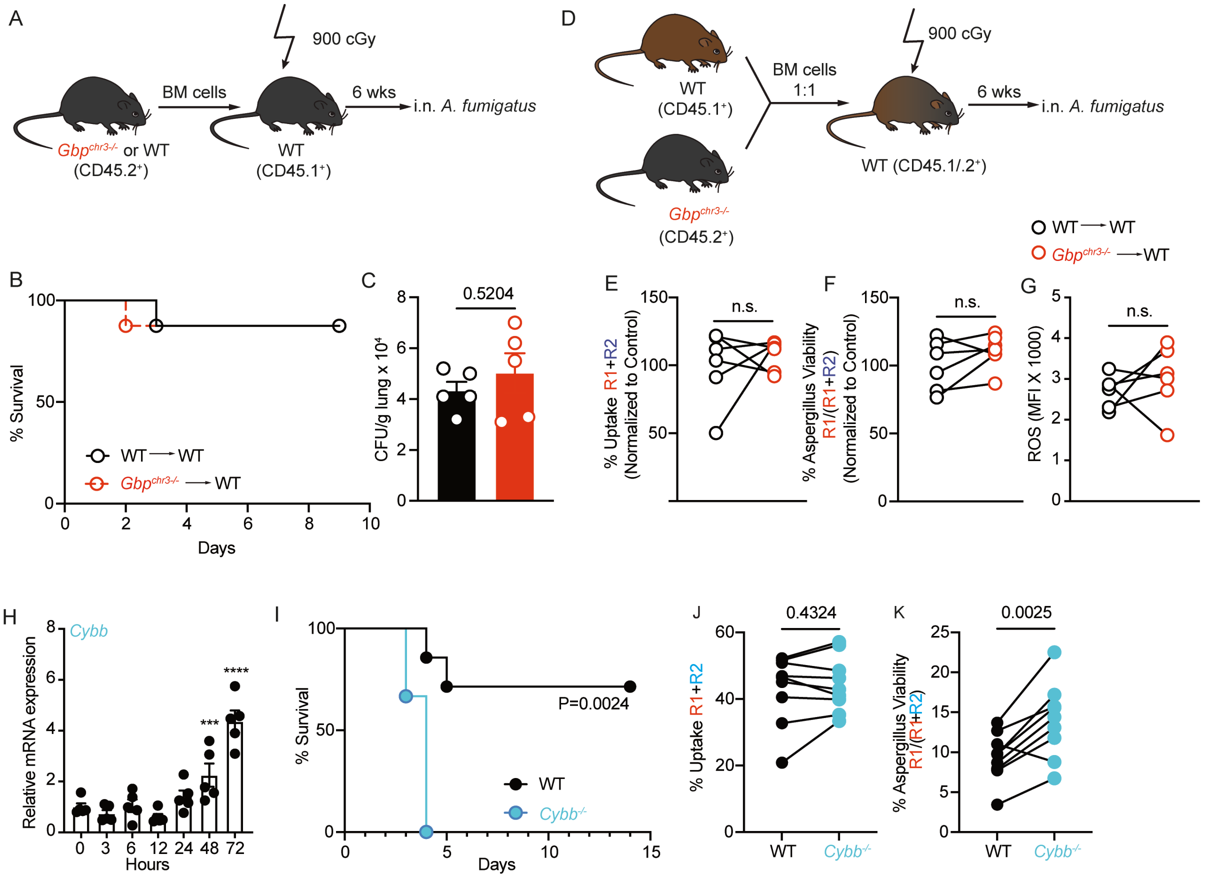

Figure S5. Related to Fig. 5. STAT1-dependent guanylate-binding proteins are dispensable for the neutrophil antifungal response.

(A) Experimental scheme to generate $GBP^{chr3-/-}$ and $GBP^{chr3+/+}$ single chimeric mice. (B) Kaplan Meier survival (n = 7-8 per group), and (C) mean ± SEM lung CFU (n = 5 per group) in $GBP^{chr3-/-}$ (red symbols) and $GBP^{chr3+/+}$ (black symbols) single chimeric mice infected with 3-6 × 10^7 CEA10 conidia. (D) Experimental scheme to generate $GBP^{chr3-/-}$ and $GBP^{chr3+/+}$ mixed chimeric mice. (E and F) The plots show normalized neutrophil (E) conidial uptake (R1 + R2) ± SEM and (F) conidial viability (R1/ (R1 + R2) ± SEM in lung neutrophils isolated from $GBP^{chr3-/-}$ (red symbols) and $GBP^{chr3+/+}$ (black symbols) mixed bone marrow chimeric mice (n = 6 per group). (G) Mean ± SEM neutrophil ROS production in neutrophils isolated from $GBP^{chr3-/-}$ (red symbols) and $GBP^{chr3+/+}$ (black

symbols) mixed bone marrow chimeric mice (n=6 per group). (H) *Cybb* gene expression in neutrophils isolated from *Stat1*$^{-/-}$ (purple symbols) and *Stat1*$^{+/+}$ (black symbols) mice. Gene expression as determined by qRT-PCR (n = 3 - 5 per group) (I) Kaplan Meier survival (n = 7 - 8 per group) of *Cybb*$^{-/-}$ (blue symbols) and WT control (black symbols) mice infected with 3 - 6 × 10^7 CEA10 conidia. (J and K) The plots show neutrophil (J) conidial uptake (R1 + R2) ± SEM and (K) conidial viability (R1/ (R1 + R2) ± SEM in lung neutrophils isolated from Cybb$^{-/-}$ (blue symbols) and wildtype (black symbols) mixed bone marrow chimeric mice (n = 9 per group). (B - K) Data are representative of 2 experiments and presented as mean ± SEM. Dots represent individual mice. Statistical analysis: (C) Mann-Whitney test. (E-G, J-K) Paired t test. (H) Kruskal-Wallis test for multiple comparisons.
