# Supplementary figures and images for: An IFN/STAT1/CYBB axis defines protective plasmacytoid DC–neutrophil crosstalk in Aspergillus fumigatus–infected mice
Source: J Clin Invest. 2025 Aug 5;135(20):e190107. doi: 10.1172/JCI190107 (PMC12520677; doi:10.1172/JCI190107)

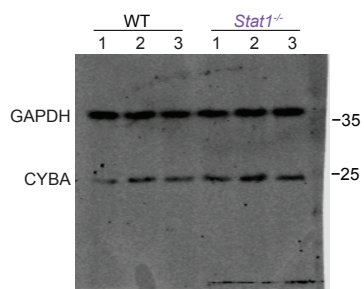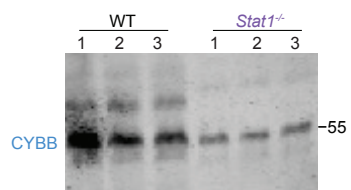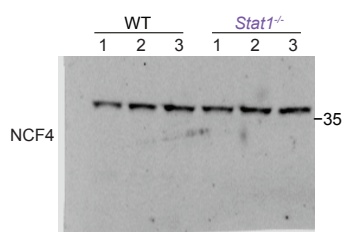

Supplement: Unedited blot and gel images [file jci-135-190107-s234.pdf]
